# A Comparison of Using Cuffed and Uncuffed Face Masks for Providing Manual Bag Ventilation in Elderly Patients with Obesity

**DOI:** 10.3390/healthcare12222214

**Published:** 2024-11-06

**Authors:** Paweł Ratajczyk, Krzysztof Wasiak, Przemysław Kluj, Tomasz Gaszyński

**Affiliations:** Department Anesthesiology and Intensive Therapy, Medical University of Lodz, 90-419 Lodz, Poland; pawel.ratajczyk@umed.lodz.pl (P.R.); krzysztof.wasiak@umed.lodz.pl (K.W.); przemyslaw.kluj@umed.lodz.pl (P.K.)

**Keywords:** cuffed and uncuffed face mask, Ambu^®^ Ultra Seal Mask, Intersurgical Eco™ Mask II, bag-valve-mask ventilation

## Abstract

Background: With the improvement of healthcare, the number of elderly individuals, including those with obesity, is increasing. The accumulation of various ventilation problems associated with the use of face masks in both these patient groups can pose a challenge even for an experienced anesthesiologist. The main aim of this study was to evaluate the ventilation of elderly patients with obesity using face masks, uncuffed or cuffed, and compare it with values obtained among patients with obesity who are not elderly. The secondary aim of the study was to demonstrate which of the two masks tested is better for elderly patients with android and gynoid obesity. Methods: This study was conducted at University Clinical Hospital No. 1 in Lodz among 108 patients with obesity, 50 elderly and 58 non-elderly. Patients’ BMIs ranged from 35.0 to 59.0. For the study, the uncuffed Intersurgical Eco Mask II and cuffed Ambu Ultra Seal face masks were used. Expiratory tidal volume and leakage obtained during the use of both types of masks were examined. The obtained data were analyzed using the Kolmogorov–Smirnov test and supplemented with Wilcoxon test values. Results: In elderly patients with obesity, especially those with gynoid obesity, the use of the Intersurgical Eco Mask II is associated with better ventilation parameters than the Ambu Ultra Seal mask. Only in the case of elderly patients with android obesity did the use of the Ambu Ultra Seal mask yield similar results to the Intersurgical Eco Mask II. Conclusions: Uncuffed face masks provide better ventilation parameters during manual bag ventilation in elderly patients with obesity.

## 1. Introduction

With the increasing number of individuals over the age of 60, which according to the WHO definition marks the beginning of old age, the aging of society poses a global challenge for world health protection. This is particularly significant because by 2050, these individuals will constitute approximately 21.8% of the total population [1]. As a result of this ongoing process, the age of operated patients and the number of patients undergoing procedures under general anesthesia are increasing. Today, there are patients undergoing surgeries who, just ten years ago, would have been considered “too old for surgery” [2]. It is estimated that older individuals require surgery four times more often than the rest of the population [1,3]. In addition to general changes in the bodies of elderly patients, there are also local changes in the respiratory tract affecting the effectiveness of general anesthesia, particularly face mask ventilation during induction. These changes include dentition loss and atrophy of the orbicularis oris muscle and collagen tissue [4,5,6,7]. All these factors contribute to the difficulty of face mask ventilation in this patient group [4]. Thus, in the literature, some authors recommend not removing artificial dental prostheses [8], while others advocate for the use of oropharyngeal airways and special tapes to secure face masks to the patient’s face [9]. Xue suggests using a larger face mask in this patient group and positioning it on the lower lip of the patient so that the patient’s chin and cheeks fit entirely within the mask [10]. Some believe that using a smaller mask size yields better results [11]. According to Crooke, placing the caudad end of the mask between the lower lip and the alveolar process reduces air leakage because a good seal is formed around a significant portion of the mask [12]. He also suggests placing moistened gauze of appropriate size on collapsed cheeks in the case of leakage to improve mask contact with the patient’s skin [9]. The existence of various techniques aimed at improving the sealing of face masks indicates that this is a challenging procedure that requires considerable experience from the anesthesiologist [4]. This situation may further deteriorate if civilization-related diseases and pathologies such as obesity are superimposed on old age. According to WHO (World Health Organization) data from 2008, over 10% of adults worldwide are obese, with a BMI > 30 kg/m^2^, and by 2030, this number may increase to 38% [9]. These patients exhibit changes in the respiratory tract, such as a narrow oral fissure, a thick short neck, a large tongue, excessive soft tissue around the larynx, and anterior displacement of its entrance, all of which hinder ventilation and intubation. The accumulation of adipose tissue around the ribs, diaphragm, and abdomen and excess adipose tissue in the abdominal cavity and chest wall reduces lung volume, exacerbating respiratory complications [13,14]. Fat deposits directly affect respiratory function, impeding diaphragmatic movement, reducing lung and chest wall compliance, and increasing resistance in the lower airways [14]. Patients with morbid obesity are not a homogeneous group of patients, as different ventilation problems can be expected in patients with android and gynoid obesity [15,16,17,18]. All these factors contribute to the difficulty of face mask ventilation in this patient group [19,20,21,22,23,24]. Many authors recommend using positive end-expiratory pressure (PEEP) during the induction of anesthesia to prevent atelectasis and extend the safe apnea time [25,26]. To generate PEEP, a tight seal between the face mask and the patient’s face is necessary, which can be very challenging in elderly patients with obesity [21,22,25].

In our study, we aimed to assess which face mask—the cuffless Eco Mask II by Intersurgical (Wokingham, UK) or the cuffed Ultra Seal Mask by Ambu (Ballerup, Denmark)—is more effective for ventilation in elderly patients with obesity. There is a lack of data in the literature regarding the use of face masks in this patient group.

## 2. Materials and Methods

### 2.1. Materials

In the study, we used the uncuffed Intersurgical Eco™ Mask II face mask ( Wokingham, UK) and the cuffed Ambu^®^ Ultra Seal Mask face mask (Ballerup, Denmark).

The Intersurgical Eco Mask II features a transparent flexible shell and a lining made of polypropylene and thermoplastic elastomer, which ensures excellent patient visibility and a seal-tight fit. It is produced in four sizes for adult patients. To facilitate identification, each size is marked with a color-coded ring. The orange color indicates size 5 (large) and the green color indicates size 4 (medium), which were the sizes used in this study for the patients.

The Ambu Ultra Seal Mask is made of polyvinyl chloride and has a soft, anatomically shaped cuff that allows for precise sealing with minimal pressure. The transparent dome provides easy observation of the patient’s condition during use. It is available in seven sizes, of which three sizes (from 4 to 6) are dedicated to adults. It features color coding for quick and easy size identification, where blue corresponds to size 5 (large) and red to size 4 (medium), and these sizes were used in this study. These are sample face masks. Patients who are elderly or have obesity are difficult to ventilate with face masks, so we are looking for the best solution.

### 2.2. Study Settings

**Figure 1:**
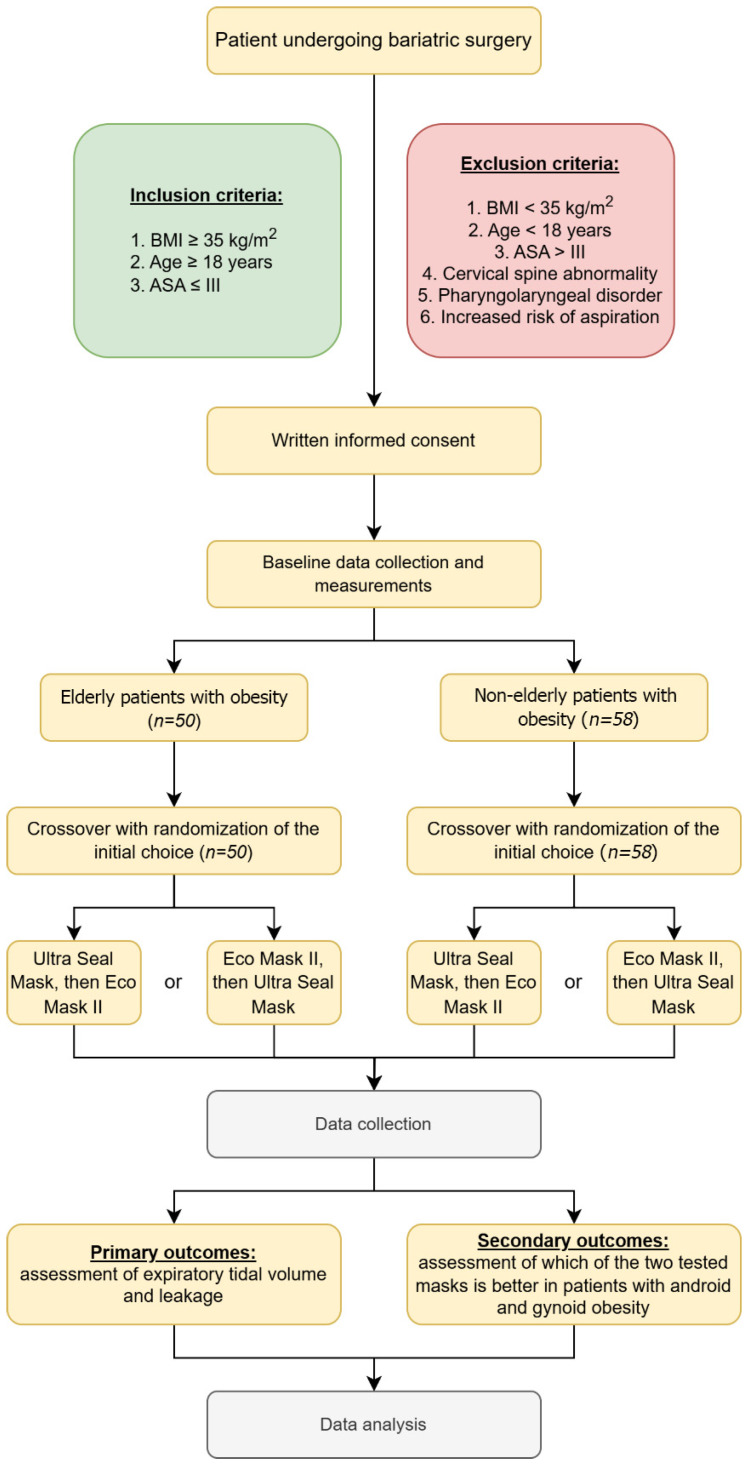
Flow chart of the present study.

This study was approved by the Bioethics Committee of the Medical University of Lodz (number RNN/103/22/KE dated 10 May 2022) and registered in Trial Registration (number NCT06219460). It was performed in the Central Operating Block of Norbert Barlicki University Teaching Hospital No. 1 in Lodz in the period from September 2022 to December 2023. Active ventilation with the assessment of expiratory volume and current leakage was performed by anesthesiologists with over 20 years of experience in anesthetizing patients with obesity. The conducted study is a randomized, controlled, blinded crossover trial with a parallel control group. The sequence in which face masks were used was randomly chosen using sealed non-transparent envelopes. The strategy of locked randomization was generated using the Randomizer program (randomizer.org). The flow diagram is presented in Figure 1.

The study included adult patients (over 18 years old) with a BMI ≥ 35 and ASA ≤ III, qualified for elective bariatric procedures under general anesthesia with endotracheal intubation, who expressed informed, voluntary consent to participate in the study. The patients were divided into two study groups: the proper group, patients of advanced age (at least 60 years old), and the control group, patients of non-advanced age (below 60 years old). Patients with a BMI < 35, ASA IV and above, with cervical spine injuries, with vascular changes in the CNS and other parts of the body, with confirmed airway pathology, after surgeries of the oral cavity, pharynx, and larynx, and those who did not consent to participate in the study were excluded from the study. Patients’ weight, height, and BMI were measured. All obtained data were anonymized.

After transporting the patient to the operating room, the envelope was opened, and the patient was qualified for the first active ventilation using one or the other face mask. During anesthesia induction, patients were placed in a supine position with the head and torso elevated at a 25-degree angle on a positioning pad. Standard anesthesia monitoring was applied: HR (heart rate), NIBP (non-invasive blood pressure), and SpO_2_ (oxygen saturation). Patients who qualified for the study underwent a standardized technique of anesthesia induction: denitrogenation with 100% oxygen for 3 min., FNT (Fentanyl) 1.5 μg/kg i.v., propofol 2.5 mg/kg i.v., and rocuronium bromide 0.6 mg/kg i.v. after confirming adequate ventilation by the face mask. Bag-mask ventilation with 100% oxygen and 2% inhaled sevoflurane was continued for 3 min. After achieving full neuromuscular blockade, confirmed by the loss of the entire train of four responses using a peripheral nerve stimulator (Innervator Constant Current Peripheral Nerve Stimulator, Fisher & Paykel Health Care System, Auckland, New Zealand), the investigator proceeded to start the study using the first randomly selected face mask. Measurements were taken after eliminating total neuromuscular conduction as a potential cause of increased resistance. During measurements, the studied face mask was continuously held with one hand using the C-E grip technique. This was important because in pre-hospital conditions, this technique allows the rescuer to independently provide manual ventilation using a self-expanding bag without the need for involvement of a second team member or an outside person. The type of mask was changed after four consecutive measurements for each mask type. To objectify the measurement, i.e., to achieve the same tidal volume introduced into the patient’s airways, ventilation with the anesthesia machine’s respirator in volume-controlled mode was applied. The measurement was taken for half a minute with unchanged anesthesia machine settings: VT = 500 mL, f = 12 breaths/min, FiO_2_ 100%, and 2% inhaled sevoflurane. The first four values of the obtained expiratory volume were recorded. Then, the study was repeated with the participation of the second tested face mask with the same anesthesia machine settings. After taking four expiratory volume measurements, the patient was intubated, connected to the anesthesia machine, and the surgical procedure continued. Expiratory tidal volume was measured (which, for the study design, was assumed to be equal to the inhaled volume), and the tightness obtained by the masks was assessed by comparing the delivered respiratory volume (500 mL) with the exhaled volume, i.e., the leakage generated by each mask was evaluated. During each mask test, four expiratory volume measurements were recorded, the mean expiratory volume values for each mask were calculated, and leakage values were calculated as the difference between the delivered and returned volume. Based on the results obtained, a summary of the data was made, which was entered into a Microsoft Office Excel spreadsheet and subjected to statistical analysis.

Because the gynoid and android types of obesity may affect the effectiveness of bag-mask ventilation due to different fat tissue distributions, the study group was divided by sex. The simplified assumption was made that the gynoid obesity type is most common in females, while the android obesity type is most common in males.

An assessment of expiratory volume and leakage during the ventilation of patients of advanced age and with obesity with the Eco Mask II and Ultra Seal Mask masks and a comparison of their values obtained during the ventilation of patients of non-advanced age and with obesity was the primary goal of the study.

The secondary objective of the study was to demonstrate which of the two tested masks is better in elderly patients with android and gynoid obesity.

### 2.3. Safety Conditions

In addition to experienced personnel, we had at our disposal all accessible devices for intubation in patients with standard and so-called difficult airways. We had equipment and experience in anesthetizing patients with different degrees of obesity confirmed by publications in many journals. Also, the set containing supraglottic airways, videolaryngoscopes, and intubating stylets for the so-called difficult airways was prepared. The second experienced anesthesiologist supervised the proceeding of study, evaluating the accordance with study protocol and watching over the patient’s safety. Sugammadex was also prepared for the rapid reversal of neuromuscular blockade in case of unsuccessful ventilation and intubation of the patient.

### 2.4. Statistical Analysis

Our aim was to assess active ventilation in patients with obesity using the Intersurgical Eco Mask II and Ambu Ultra Seal Mask based on expiratory volume and leakage produced. Expiratory volume was evaluated during four measurements using both masks, the mean expiratory volume when using each mask, leakage values for 4 measurements, and the mean leakage when using each mask. First, the assumption of normal distribution of the analyzed variables was verified. The Kolmogorov–Smirnov test was employed for this purpose. Since statistically significant deviations from the normal distribution were found for all analyzed variables, subsequent analyses were conducted based on non-parametric tests of statistical significance. The main part of the statistical analysis concerns the comparison of expiratory volume and leakage obtained during ventilation using the Intersurgical Eco Mask II and the Ambu Ultra Seal Mask. As both types of masks were used for ventilation in each patient, and the distribution of analyzed variables deviated from normal distribution, the Wilcoxon test was used to verify the statistical significance of differences between the two types of masks.

## 3. Results

### 3.1. Characteristics of Patients Included in the Study

Fifty elderly patients with obesity and fifty-eight non-elderly patients with obesity were included in the study. In the group of elderly patients with obesity, there were 12 men and 38 women. In the group of non-elderly patients with obesity, there were 20 men and 38 women. The demographic data of the studied groups are presented in Table 1. In our study, all females had the gynecoid type of obesity and males had the android type.

### 3.2. Primary Objective of the Study

#### 3.2.1. Elderly Patients with Obesity

**Figure 2:**
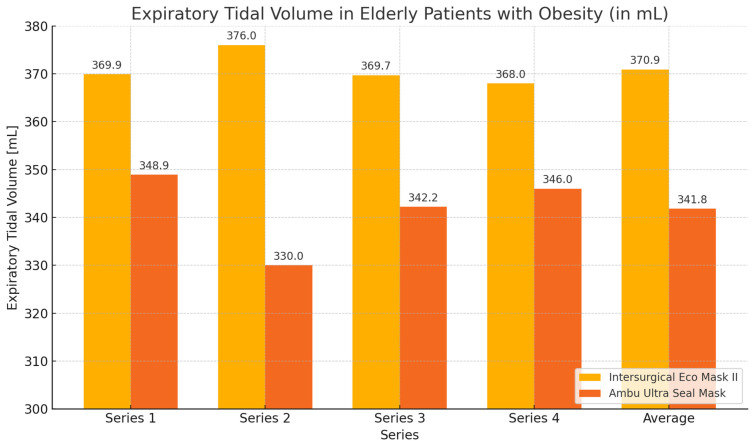
Expiratory volume chart obtained during ventilation of elderly patients with obesity with Eco Mask II and Ultra Seal Mask.

**Figure 3:**
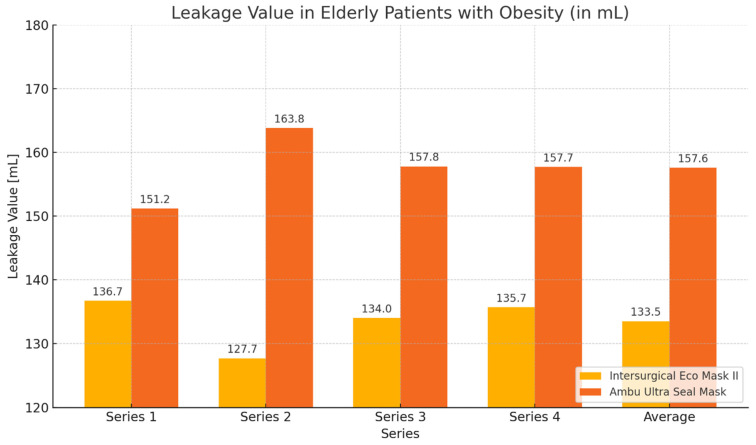
Leakage values chart obtained during ventilation of patients with Eco Mask II and Ultra Seal Mask.

In the group of elderly patients with obesity, higher expiratory volume values and lower leakage values were achieved when using the Eco Mask II compared to the Ultra Seal Mask. The obtained results were statistically significant for the mean expiratory volume, mean leakage value, and the volume and leakage obtained during the second measurement (Table 2, Figure 2 and Figure 3).

#### 3.2.2. Non-Elderly Patients with Obesity

**Figure 4:**
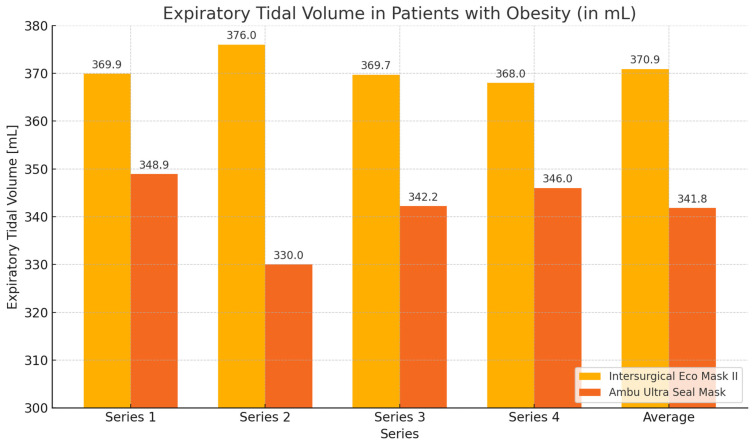
Graph showing values of expiratory volume and leakage obtained during ventilation of non-elderly patients with obesity using Eco Mask II and Ultra Seal Mask.

**Figure 5:**
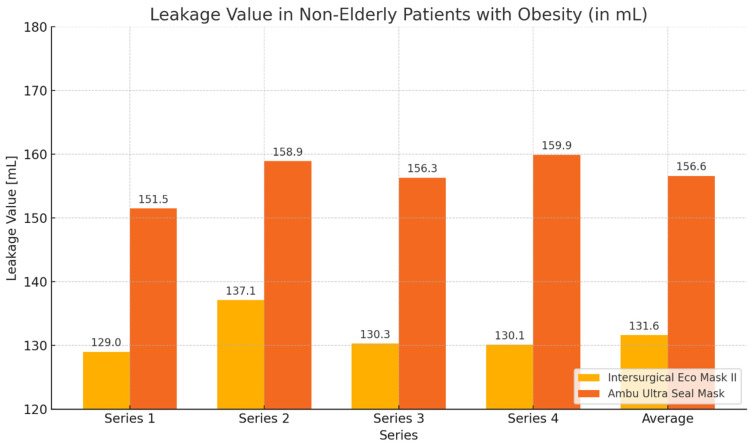
Graph showing leakage values during ventilation of non-elderly patients with obesity using Eco Mask II and Ultra Seal Mask.

In the group of non-elderly patients with obesity (control group), similar to the elderly patients with obesity group, greater expiratory volume values and smaller leakage values were obtained during ventilation with the Eco Mask II. However, the obtained results were not statistically significant (Table 3, Figure 4 and Figure 5).

### 3.3. Secondary Objective of the Study

#### 3.3.1. Elderly Patients with Gynoid Type of Obesity

**Figure 6:**
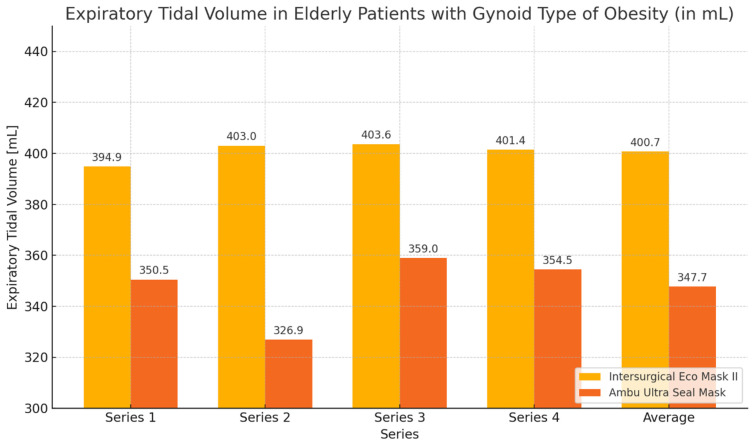
Chart of tidal volume during ventilation of elderly patients with gynoid obesity using Eco Mask II and Ultra Seal Mask.

**Figure 7:**
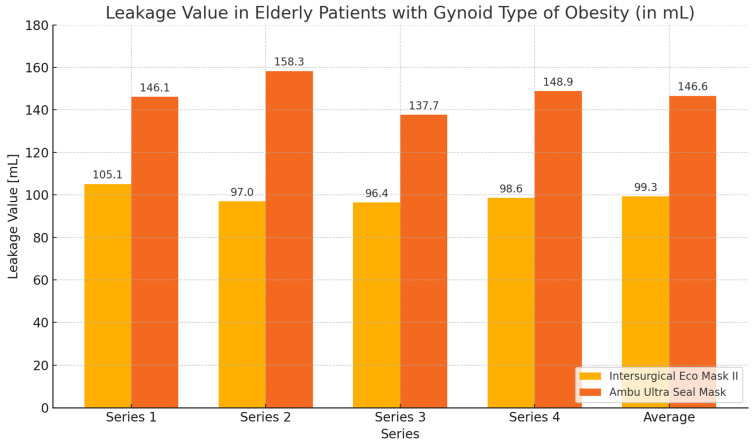
Chart of leakage values during ventilation of elderly patients with gynoid obesity using Eco Mask II and Ultra Seal Mask.

During the ventilation of elderly patients with gynoid obesity, higher tidal volume values and lower leakage values were achieved when using the Eco Mask II compared to the Ultra Seal Mask. These differences were statistically significant for the average tidal volume values and average leakage, as well as for most of the measurements except for series 1 (Table 4, Figure 6 and Figure 7).

#### 3.3.2. Non-Elderly Patients with Gynoid Obesity

**Figure 8:**
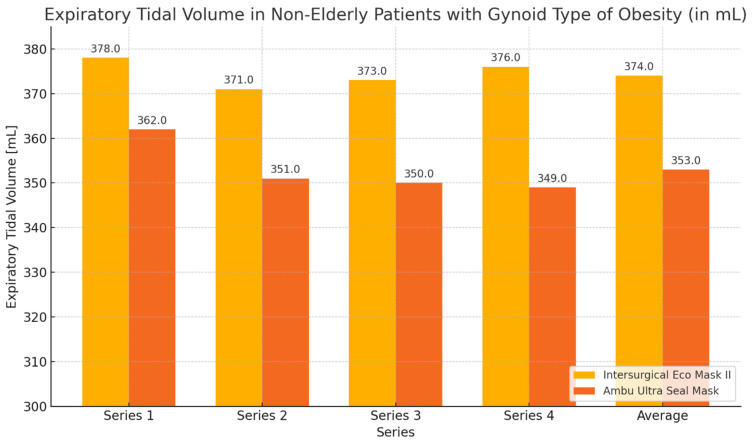
Expiratory volume chart during ventilation of non-elderly patients with gynoid obesity using Eco Mask II and Ultra Seal Mask.

**Figure 9:**
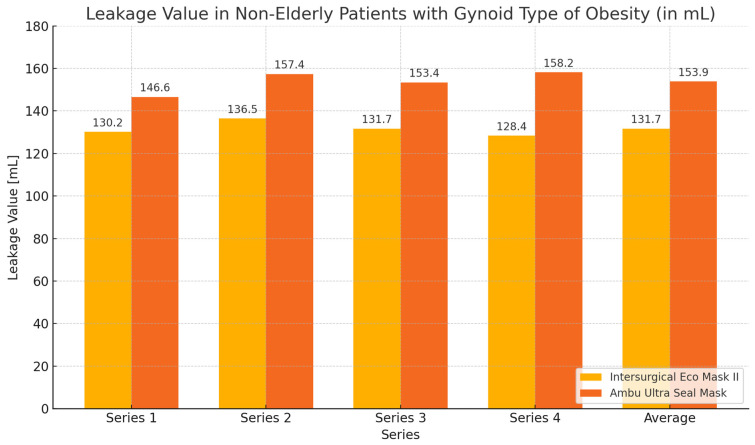
Leakage values chart during ventilation of non-elderly patients with gynoid obesity using Eco Mask II and Ultra Seal Mask.

During the ventilation of non-elderly patients with gynoid obesity using the Eco Mask II and Ultra Seal Mask, higher expiratory volume values and lower leakage values were obtained with the Eco Mask II. These differences were not statistically significant (Table 5, Figure 8 and Figure 9).

#### 3.3.3. Elderly Patients with Android Obesity

**Figure 10:**
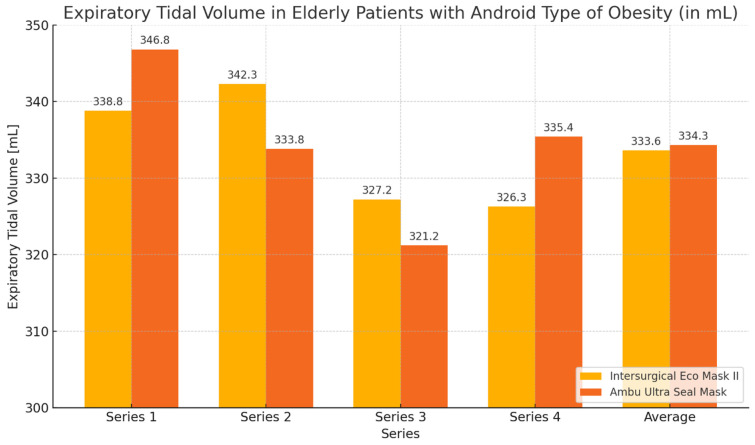
Expiratory volume graph during ventilation of elderly patients with android obesity using Eco Mask II and Ultra Seal Mask.

**Figure 11:**
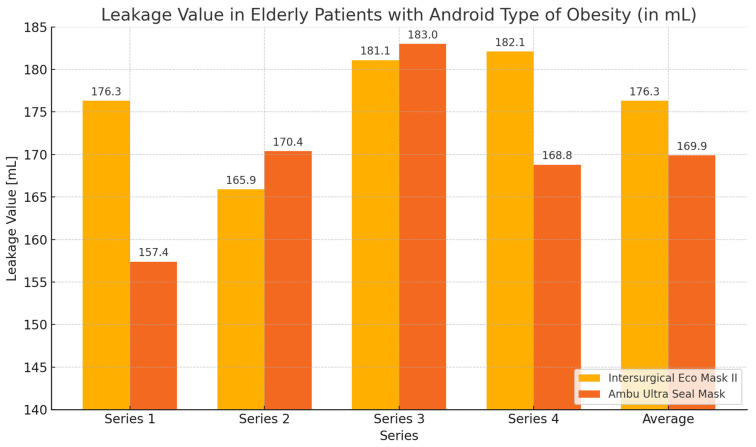
Leakage values graph during ventilation of elderly patients with android obesity using Eco Mask II and Ultra Seal Mask.

During the ventilation of elderly patients with android obesity, the average expiratory volume values obtained with the Ultra Seal Mask were slightly higher than those with the Eco Mask II. Regarding the average leakage values, the Ultra Seal Mask exhibited greater tightness, characterized by lower leakage. Analyzing the individual series of the study, it was observed that with the Ultra Seal Mask, a higher expiratory volume was achieved in series 1 and 4. Conversely, when using the Eco Mask II, higher expiratory volume values were obtained during series 2 and 3. The leakage values showed corresponding opposite trends when using both masks. However, the observed differences in expiratory volume and leakage values were not statistically significant (Table 6, Figure 10 and Figure 11).

#### 3.3.4. Non-Elderly Patients with Android Obesity Type

**Figure 12:**
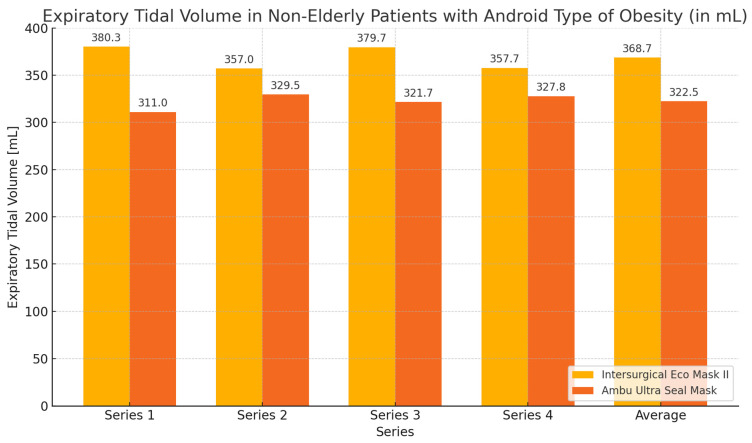
Expiratory volume chart during ventilation of non-elderly patients with android obesity using Eco Mask II and Ultra Seal Mask.

**Figure 13:**
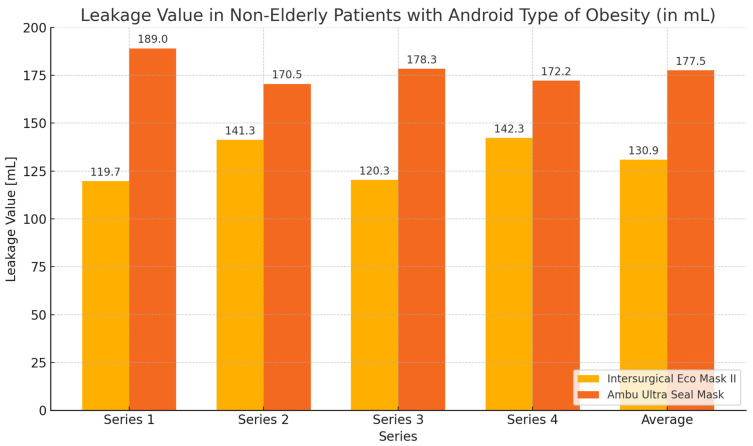
Leakage value chart during ventilation of non-elderly patients with android obesity using Eco Mask II and Ultra Seal Mask.

In this group of subjects, the highest values of expiratory volume and the greatest tightness—the smallest leakage—were obtained during ventilation with the Eco Mask II compared to the results obtained when using the Ultra Seal Mask. However, these differences were not statistically significant (Table 7, Figure 12 and Figure 13).

## 4. Discussion

In the available literature, there are numerous studies addressing the issue of ventilating patients using a face mask during anesthesia induction with incomplete or total dentition loss, as is the case in elderly patients [1,2,3,4,8,9,10,11,12,27]. Various techniques, often sophisticated, facilitating adequate sealing between the mask and the patient’s face during this procedure have also been described [8,9,10,11,12]. Similarly, a considerable number of publications describe ventilation problems with a face mask in patients with different degrees of obesity [18,19,22,23,25,26]. Different ventilation techniques suitable for this patient group are also discussed [28,29,30]. However, there is a lack of publications addressing ventilation in elderly patients with obesity, a population in which ventilation issues present in both these groups separately could become apparent. The problems associated with excessive fat accumulation in patients with obesity overlap here with the loss of collagen fibers in the skin and tissues, incomplete or complete dentition loss, and the accumulation of pharyngeal fat (independent of BMI) in elderly patients [4,5,6,7].The combination of these two types of changes in our study population may exacerbate ventilation problems with a face mask present in each of these groups separately. Difficulty with ventilation with a face mask is observed in 12% of patients with incomplete dentition, as seen among elderly patients, while in patients with obesity, this percentage may be as high as 17.2% [4,19]. However, this may be reduced due to a previously unmentioned coincidence with increased fat tissue accumulation and physiologic changes occurring in elderly patients affecting airway patency and the effectiveness of face mask sealing. Patients with obesity may also face different types of fat tissue accumulation present in individuals with android and gynoid obesity, which also affect the seal obtained by the face mask and the ventilation efficiency [16,17,18].

In our study, we compared the usability of two types of masks, the cuffless Intersurgical Eco Mask™ II and the cuffed Ambu^®^ Ultra Seal Mask, in elderly patients with obesity (with android and gynoid obesity). The control group consisted of patients with obesity who were not elderly. In our study, better, statistically significant differences in tidal volume and leakage were obtained for the Eco Mask II compared to the Ultra Seal Mask. In the group of non-elderly patients with obesity, better tidal volume and lower leakage values were also achieved with the cuffless mask compared to the cuffed mask, but these values were not statistically significant. When comparing the ventilation effectiveness of elderly patients with gynoid obesity, statistically significant better tidal volume and lower leakage values were achieved when using the Eco Mask II compared to the Ultra Seal Mask. A similar situation occurred when both masks were used among non-elderly patients, but these values were not statistically significant. In elderly patients with android obesity, the cuffed mask, the Ultra Seal Mask, was only slightly better—on the verge of equality—than the cuffless mask. This was the first situation in our study where the cuffed mask was found to provide better ventilation parameters than the cuffless mask. When used in non-elderly patients, again, as in the entire study, the cuffless mask was superior. However, the results obtained were not statistically significant. In our study, there was no need to use additional techniques to improve sealing between the face mask and the patient’s skin, such as those described in the literature—tape securing the mask to the patient’s face, moistened gauzes, larger-sized face masks in elderly patients with obesity— and even more so among younger patients [8,9,10,11,12,27,28,31]. This may suggest that additional fat tissue accumulation around the oral cavity and face in elderly patients with obesity somewhat counteracts the loss of collagen fibers and the effect of missing or incomplete dentition on face mask sealing and airway patency. However, it should be noted that in our study, there were no completely edentulous patients, and therefore, such conclusions should be drawn cautiously as a hypothesis requiring further investigation. Moreover, statistically significant better ventilation parameters and lower leakage were obtained in patients with gynoid obesity, in whom fat tissue accumulates more often in the abdominal area than in the upper body. The superiority of the cuffless mask likely results from the silicone material used and the soft ring of this mask, which adheres more easily and tightly to the facial skin due to body temperature. Similar conclusions were drawn by Gaszyński, who evaluated two cuffed and two cuffless masks in his study [32]. This was a study on a manikin model, where, according to many authors, achieving sealing is more difficult than in real-life conditions. Ball also obtained better sealing with the Intersurgical Eco Mask II, conducting his study on healthy volunteers with a protocol similar to that used in our study [33].

Nandalan et al. concluded in their work that an antistatic rubber face mask is superior to two plastic masks during the routine induction of general anesthesia based on various measured parameters. The authors also hope that this will encourage manufacturers to improve the design of disposable face masks to simulate an antistatic rubber face mask [34].

## 5. Conclusions

Based on the results of our study, it appears that in elderly patients with obesity, especially those with gynoid obesity, the use of the Intersurgical Eco Mask II is associated with better ventilation parameters than the Ambu Ultra Seal mask. Only in elderly patients with android obesity does the use of the Ambu Ultra Seal mask yield similar results to the Intersurgical Eco Mask II. Recommending the wider use of the Intersurgical Eco Mask II in elderly patients with obesity requires further clinical research on a larger study group.

## 6. Limitations

This study has several limitations. Firstly, the group of patients with android obesity was relatively small. Secondly, the study group consisting of 50 elderly patients with obesity may be too small to provide unequivocal recommendations for the use of a particular face mask in these patients, especially in individuals with severe obesity. The V-E grip, which is more useful when ventilating patients with obesity, was not used to hold the face mask during this study. This study was conducted by anesthesiologists with more than 20 years of experience in the anesthesia of patients with obesity, and their results may differ from those of less-experienced clinicians. Another limitation is the use of only one sample mask in each group of cuffed and uncuffed face masks.

## Figures and Tables

**Table 1:** Clinical characteristics of patients included in the study.

		*n*	M	SD	Min	Max
Age	Total	108	44.3	11.9	21	75
	Females	76 (70.4%)	41.7	11.0	21	74
	Males	32 (29.6%)	50.6	11.7	21	75
	Elderly	50 (46.3%)	66.3	4.6	60	75
	Females	38 (76%)	67.5	4.4	60	74
	Males	12 (14%)	65.7	3.7	60	75
	Non-Elderly	58 (53.7%)	40.0	11.3	21	59
	Females	38 (65.5%)	38.5	11.7	21	59
	Males	20 (34.5%)	47.5	11.6	21	59
Height	Total	108	169.2	7.1	155	186
	Females	76 (70.4%)	166.0	5.1	155	176
	Males	32 (29.6%)	176.5	5.5	160	186
Body	Total	108	122.9	1.5	90	170
mass	Females	76 (70.4%)	120.5	16.0	90	162
	Males	32 (29.6%)	128.7	16.3	104	170
BMI	Total	108	43.2	5.7	35.0	59.8
	Females	76 (70.4%)	44.0	5.6	35.1	59.8
	Males	32 (29.6%)	41.3	4.8	35.0	54.7

*n*—number of individuals; M—mean value; SD—standard deviation; min—minimum value; max—maximum value.

**Table 2:** Expiratory volume values and leakage obtained in the group of elderly patients with obesity during ventilation with the Eco Mask II and Ultra Seal Mask. M—mean value; SD—standard deviation; Z—Wilcoxon test value; *p*—statistical significance. Bold means *p* < 0.05.

	Mask					
	Intersurgical Eco Mask II		Ambu Ultra Seal Mask			
Variables	M	SD	M	SD	Z	*p*
Expiratory volume 1	369.9	118.4	348.8	118.20	−1.6	0.106
Leakage 1	136.7	129.3	151.1	120.81	−1.5	0.140
Expiratory volume **2**	**376.0**	**114.4**	**329.9**	**126.68**	**−2.6**	**0.008**
Leakage **2**	**127.6**	**118.7**	**163.8**	**123.78**	**−2.4**	**0.018**
Expiratory volume 3	369.7	120.9	342.2	118.06	−1.9	0.052
Leakage 3	134.0	126.4	157.8	121.27	−1.8	0.064
Expiratory volume 4	367.9	124.1	346.0	120.29	−1.4	0.161
Leakage 4	135.7	129.6	157.7	118.82	−1.4	0.155
Expiratory volume − mean	**370.9**	**115.1**	**341.7**	**109.75**	**−2.3**	**0.022**
Leakage − mean	**133.5**	**121.6**	**156.9**	**113.95**	**−2.1**	**0.035**

**Table 3:** Values of expiratory volume and leakage during ventilation of non-elderly patients with obesity using Eco Mask II and Ultra Seal Mask. M—mean value; SD—standard deviation; Z—Wilcoxon test value; *p*—statistical significance.

	Mask					
	Intersurgical Eco Mask II		Ambu Ultra Seal Mask			
Variables	M	SD	M	SD	Z	*p*
Expiratory volume 1	378.9	103.5	358.1	99.8	−1.5	0.141
Leakage 1	128.9	107.2	151.5	110.5	−1.5	0.136
Expiratory volume 2	370.6	105.4	350.7	99.9	−1.4	0.157
Leakage 2	137.1	107.8	158.9	104.1	−1.5	0.131
Expiratory volume 3	377.5	102.0	353.3	105.1	−1.5	0.138
Leakage 3	130.3	109.4	156.3	109.8	−1.5	0.128
Expiratory volume 4	378.5	106.0	349.8	91.8	−1.7	0.091
Leakage 4	130.1	110.1	159.8	97.7	−1.7	0.087
Expiratory volume − mean	376.4	97.3	352.9	90.4	−1.6	0.115
Leakage − mean	131.6	101.9	156.6	97.4	−1.6	0.114

**Table 4:** Values of tidal volume and leakage during ventilation of elderly patients with gynoid obesity. M—mean value; SD—standard deviation; Z—Wilcoxon test value; *p*—statistical significance.

	Mask					
	Intersurgical Eco Mask II		Ambu Ultra Seal Mask			
Variables	M	SD	M	SD	Z	*p*
Expiratory volume 1	394.9	97.2	350.5	123.0	−2.1	0.038
Leakage 1	105.1	97.2	146.1	125.5	−1.9	0.053
Expiratory volume 2	402.9	99.9	326.9	137.0	−2..1	0.007
Leakage 2	97.0	99.9	158.3	127.4	−2.4	0.017
Expiratory volume 3	403.6	100.9	358.9	125.7	−2.3	0.021
Leakage 3	96.4	100.9	137.7	126.9	−2.2	0.030
Expiratory volume 4	401.4	104.6	354.5	136.4	−2.3	0.020
Leakage 4	98.6	104.6	148.9	132.2	−2.3	0.019
Expiratory volume − mean	400.7	95.3	347.7	118.0	−2.7	0.007
Leakage − mean	99.3	95.3	146.6	121.5	−2.5	0.014

**Table 5:** Values of expiratory volume and leakage during ventilation of non-elderly patients with gynoid obesity. M—mean value; SD—standard deviation; Z—Wilcoxon test value; *p*—statistical significance.

	Mask					
	Intersurgical Eco Mask II		Ambu Ultra Seal Mask			
Variables	M	SD	M	SD	Z	*p*
Expiratory volume 1	378.7	106.9	364.2	101.7	−1.1	0.254
Leakage 1	130.2	110.9	146.6	114.1	−1.2	0.245
Expiratory volume 2	372.4	106.0	353.9	99.8	−1.4	0.152
Leakage 2	136.5	109.1	157.4	104.7	−1.5	0.130
Expiratory volume 3	377.2	104.1	357.5	106.6	−1.2	0.248
Leakage 3	131.7	112.2	153.4	112.2	−1.2	0.229
Expiratory volume 4	381.3	105.9	352.6	93.4	−1.6	0.104
Leakage 4	128.4	110.8	158.2	100.3	−1.6	0.102
Expiratory volume − mean	377.4	98.3	356.9	91.8	−1.4	0.157
Leakage − mean	131.7	103.6	153.9	99.9	−1.4	0.158

**Table 6:** Expiratory volume and leakage values during ventilation of elderly patients with android obesity. M—mean value; SD—standard deviation; Z—Wilcoxon test value; *p*—statistical significance.

	Mask					
	Intersurgical Eco Mask II		Ambu Ultra Seal Mask			
Variables	M	SD	M	SD	Z	*p*
Expiratory volume 1	338.7	136.3	346.7	114.5	0.01	0.999
Leakage 1	176.2	153.9	157.4	117.0	−0.03	0.976
Expiratory volume 2	342.3	124.3	333.7	115.2	−0.8	0.417
Leakage 2	165.9	130.9	170.4	121.6	−0.8	0.417
Expiratory volume 3	327.2	132.3	321.2	106.7	−0.5	0.595
Leakage 3	181.1	140.9	182.9	111.2	−0.5	0.595
Expiratory volume 4	326.2	135.7	335.4	98.4	−0.3	0.784
Leakage 4	182.1	144.4	168.7	101.4	−0.3	0.784
Expiratory volume − mean	333.6	128.4	334.3	100.4	−0.4	0.715
Leakage − mean	176.3	138.5	169.9	104.9	−0.4	0.715

**Table 7:** Values of expiratory volume and leakage during ventilation of non-elderly patients with android obesity. M—mean value; SD—standard deviation; Z—Wilcoxon test value; *p*—statistical significance.

	Mask					
	Intersurgical Eco Mask II		Ambu Ultra Seal Mask			
Variables	M	SD	M	SD	Z	*p*
Expiratory volume 1	380.3	80.6	311.0	74.5	−1.1	0.249
Leakage 1	119.7	80.6	189.0	74.5	−1.1	0.249
Expiratory volume 2	357.0	109.6	329.5	107.5	−0.3	0.753
Leakage 2	141.3	107.0	170.5	107.5	−0.3	0.753
Expiratory volume 3	379.7	93.6	321.7	94.4	−0.9	0.345
Leakage 3	120.3	93.6	178.3	94.4	−0.9	0.345
Expiratory volume 4	357.7	114.0	327.8	81.7	−0.3	0.753
Leakage 4	142.3	114.0	172.2	81.7	−0.3	0.753
Expiratory volume − mean	368.7	97.6	322.5	79.2	−0.7	0.463
Leakage − mean	130.9	96.9	177.5	79.2	−0.7	0.463

## Data Availability

Data will be made available on request due to privacy or ethical restrictions.
